# Steps to Take to Enhance Gait Stability: The Effect of Stride Frequency, Stride Length, and Walking Speed on Local Dynamic Stability and Margins of Stability

**DOI:** 10.1371/journal.pone.0082842

**Published:** 2013-12-13

**Authors:** Laura Hak, Han Houdijk, Peter J. Beek, Jaap H. van Dieën

**Affiliations:** 1 Research Institute MOVE, Faculty of Human Movement Sciences, VU University Amsterdam, Amsterdam, The Netherlands; 2 Heliomare Rehabilitation Centre, Wijk aan Zee, The Netherlands; Delft University of Technology (TUDelft), The Netherlands

## Abstract

The purpose of the current study was to investigate whether adaptations of stride length, stride frequency, and walking speed, independently influence local dynamic stability and the size of the medio-lateral and backward margins of stability during walking. Nine healthy subjects walked 25 trials on a treadmill at different combinations of stride frequency, stride length, and consequently at different walking speeds. Visual feedback about the required and the actual combination of stride frequency and stride length was given during the trials. Generalized Estimating Equations were used to investigate the independent contribution of stride length, stride frequency, and walking speed on the measures of gait stability. Increasing stride frequency was found to enhance medio-lateral margins of stability. Backward margins of stability became larger as stride length decreased or walking speed increased. For local dynamic stability no significant effects of stride frequency, stride length or walking speed were found. We conclude that adaptations in stride frequency, stride length and/or walking speed can result in an increase of the medio-lateral and backward margins of stability, while these adaptations do not seem to affect local dynamic stability. Gait training focusing on the observed stepping strategies to enhance margins of stability might be a useful contribution to programs aimed at fall prevention.

## Introduction

While substantial variance in walking speed is apparent between individuals, a more or less consistent speed is selected by individuals during steady state walking [1]. Moreover, at any given walking speed it is possible to select different combinations of stride frequency and stride length, but again individuals tend to choose a specific stride frequency and length consistently [2]. Previous studies have suggested that energy cost [3], [4] and variance thereof due to variance in anthropometrical characteristics such as leg inertia [1], [2] determine the selection of a certain gait pattern. However, optimizing energy cost might not be the sole objective when walking. Especially for people at an increased risk of falling, the choice for a certain gait pattern may be related to improving gait stability and reducing fall risk rather than minimizing energy cost. Fallers and fall-prone people often walk slower, with shorter steps and a lower step frequency than non-fallers [5], [6], [7], [8]. These differences in gait pattern, in particular the lower walking speed, are often explained as strategies to decrease fall risk [9], [10], [11], [12]. However, from such observational data, it remains unclear whether these differences in gait pattern represent a strategy to reduce the risk of falling or whether these differences in gait pattern serve other purposes, and might even coincide with an increased risk of falling.

To investigate whether gait pattern selection or adaptations could serve the purpose of decreasing fall risk, several studies have examined responses to balance perturbations during gait[13], [14], [15], [16], [17], [18]. Evidence was found that able-bodied people, but also people with a trans-tibial prosthesis[15], effectively deal with perturbations by increasing their stride frequency and decreasing their stride length, while keeping walking speed constant. These results suggest that instead of a decrease in walking speed, an increase in stride frequency and a decrease in stride length are adopted to minimize the risk of falling.

**Figure 1:**
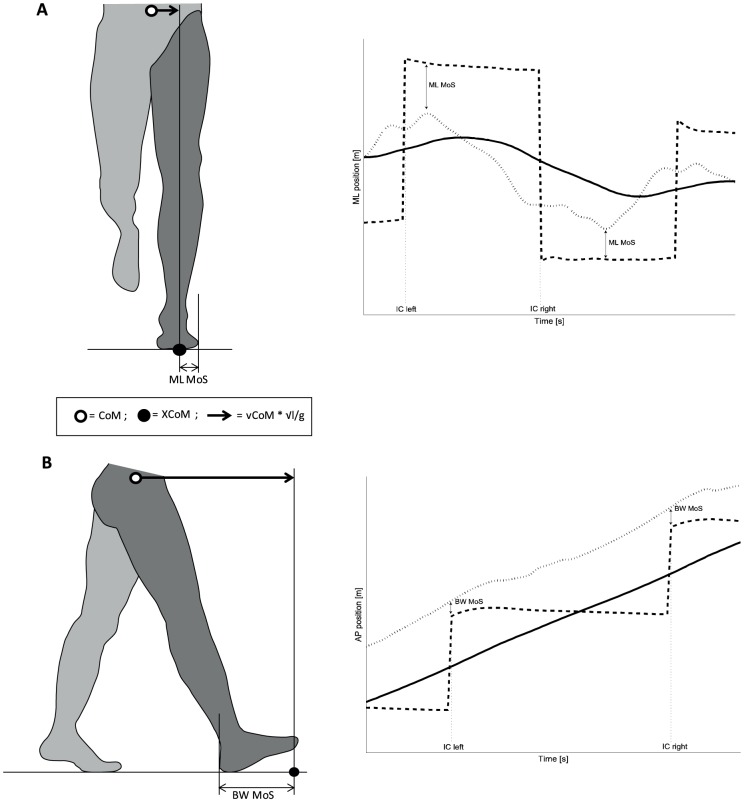
Schematic representation of the definition of the backward (BW) and medio-lateral (ML) margin of stability (MoS). **A**: The ML MoS is defined as the minimum distance in medio-lateral direction between the extrapolated centre of mass (XCoM; dotted line in the right panel) and the lateral border of the foot attained during foot-contact (solid line in the right panel). The XCoM is calculated as the position of the centre of mass (CoM; dashed line in the right panel) plus its velocity (vCoM) times a factor √(l/g), with l being the length of the pendulum (for which often the leg length is used) and g the acceleration of gravity **B**: The BW MoS is defined as the distance in anterio-posterior direction between the XCoM (dotted line in the right panel) and the posterior border of the leading foot (solid line in the right panel) at initial contact.

An alternative approach to investigate whether selection of spatio-temporal step parameters could serve the purpose of reducing fall risk is to systematically examine their effects on gait stability. In contrast to the first approach, this approach is dependent on the selected parameter to quantify gait stability or associated fall risk. The short-term Lyapunov exponent (λs), a measure of the local dynamic stability (LDS), was proposed to quantify gait stability [19] and has gained considerable support [20]. The λs quantifies the average logarithmic rate of divergence of a system after a small perturbation over a period of 0.5 or 1 stride. An increase in λs, thus implies a decrease of LDS. Previous studies indicate that the margins of stability (MoS) may provide additional information on gait stability[13], [21], [22]. The size of the MoS reflects the divergence of the centre of mass (CoM) that one can handle before an actual loss of balance occurs. The MoS is calculated as the distance between the extrapolated centre of mass (XCoM) and the limits of the base of support, in which the XCoM represents the state of the CoM taking into account both its position and velocity [21]. MoS can be calculated in medio-lateral (ML; figure 1a) and backward (BW; figure 1b) direction. A negative ML MoS will result in a deviation from the straight walking trajectory, while a negative BW MoS will result in an interruption of the forward progression. Consequently, in case of a negative MoS, a crossing step or a backward step will be necessary to prevent, respectively, a sideward or backward fall. It should be noted that an increase in BW MoS simultaneously has the disadvantage that the risk of a forward loss of balance will increase. However, while a backward loss of balance inflicts a large chance of state of the walking movement, requiring a reversal of the periodic leg movement (stepping backward) to regain balance, a forward loss of balance requires a relatively small adaption of the next step(s), like a temporary increase in step length, to recover [23]. In line with the latter, we have previously found that able-bodied subjects, but also transtibial amputees preferred to increase their BW MoS in response to ML perturbations of the walking surface[13], [15].

Several studies have investigated the effect of walking speed and the underlying step parameters on λs. Although most of these studies found that a decrease in walking speed decreased the λs, and therefore increased LDS [9], [10], [11], [24], a more recent study, in which the effect of time-series length and number of steps on λs calculation was taken into account, disputed this claim [25]. McAndrew and Dingwell [26] investigated the effect of different stride lengths at the same walking speed on λs of trunk kinematics. They expected to find lower values for λs when subjects walk with short steps. However, this appeared to be true for only the λs calculated for the medio-lateral direction, and therefore these results did not fully reconcile earlier conflicting findings on the relationship between adaptations in step length and fall risk.

In contrast with the effects of the gait pattern on LDS, the effect of adaptations of the gait parameters on the size of the ML and BW MoS can be predicted analytically. Based on the inverted pendulum behavior of the human body while walking, an increase in stride frequency is expected to increase the ML MoS [21], [23], while a decrease in stride length and an increase in walking speed is expected to increase the BW MoS [27], [28]. In agreement with these models an increase in BW MoS was indeed found when subjects walked faster or with shorter steps compared to their comfortable gait pattern [27], [28], [29].

The interpretation of the experimental results described in the previous paragraphs is, however, obscured by the fact that stride frequency, stride length, and walking speed cannot be adapted independently from each other. Consequently, the results might not only be caused by the imposed manipulation, but could also be an effect of a concomitant change in one of the other gait parameters. Therefore, a systematic analysis of the effect of stride length and frequency and their interaction, i.e., walking speed, should be conducted to reveal their independent effects on gait stability and associated fall risk.

The purpose of the current study was to investigate whether manipulations of stride frequency, stride length, and consequently walking speed, independently influence LDS expressed as λs and the ML and BW MoS. We hypothesized that λs would change in response to the imposed manipulations, but because of the large variation in results of previous studies that investigated the effect of gait pattern manipulations on λs, the direction of the change could not be predicted. With respect to the MoS, we hypothesized that the ML MoS only increases due to an increase in stride frequency [30], while BW MoS increases due to a decrease in stride length and due to an increase in walking speed [27], [28].

## Methods

### Subjects

Nine young healthy subjects (6 men and 4 women, age: 21.9±1.8 years, weight: 73.4±8.7 kg, length: 1.79±0.09 m) were included. The study was approved by the local ethical committee of the Faculty of Human Movement Sciences, VU University Amsterdam, and subjects gave written informed consent prior to their participation.

### Equipment

**Figure 2:**
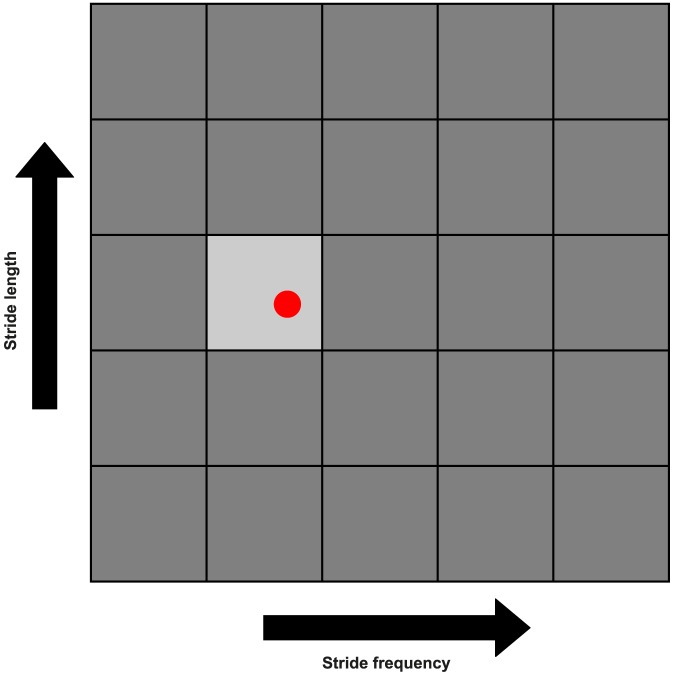
Visual feedback given during the experiment. Each cell corresponds to a certain combination of stride frequency and stride length. The middle cell corresponds to the combination of comfortable stride length and stride frequency. The percentages of comfortable stride length and stride frequency that correspond to the other cells are given in figure 3. For each experimental trial one of the cells became light gray to represent the required combination of stride length and stride frequency for that specific trial, while the red dot represented the real-time feedback on the combination of stride length and stride frequency that subjects were using.

All subjects walked on an instrumented treadmill placed in the front of a screen, which was used to give subjects real-time, visual feedback about the combination of stride frequency and stride length they were employing (Figure 2). Single infrared Light Emitting Diodes (LEDs) were attached to the lateral malleoli of the ankles and the heels of each subject, and a neoprene band with a cluster of three LEDs was attached between the left and right posterior superior iliac spines (PSIS). The LED's were used for movement registration with an active 3D movement registration system (Optotrak® Northern Digital Inc., Waterloo, Ontario, Canada).A custom made application (LabVIEW, National Instruments, Utrecht, The Netherlands) was used to calculate stride frequency and stride length during walking, based on the position of the markers attached to the ankles, and to give real time feedback about these parameters.

### Protocol

The experiment started with a pre-experimental trial. In this trial the comfortable walking speed was determined, by gradually increasing the belt speed until a comfortable walking speed was reported. Belt speed was then increased beyond the reported comfortable walking speed and subsequently gradually decreased again until a comfortable walking speed was reported. Comfortable walking speed was determined as the average of the two reported comfortable walking speeds. After the determination of comfortable walking speed, the treadmill was set at this walking speed to determine comfortable stride frequency and stride length over a period of 2 minutes.

**Figure 3:**
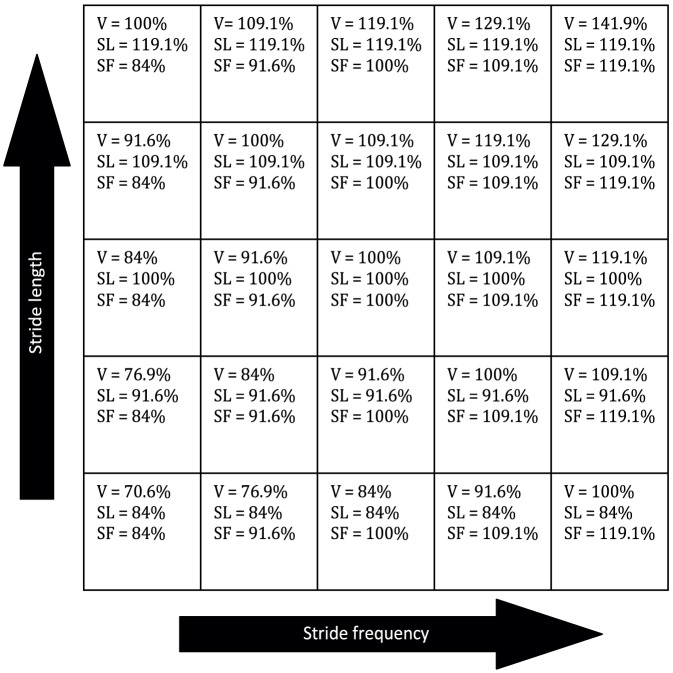
Schematic overview of the experimental conditions. (V: Walking speed; SL: Stride length, SF: Stride frequency). Percentages are percentages of respectively comfortable walking speed, stride length, and stride frequency. The rows represent the stride length manipulations and the columns the stride frequency manipulation. Note that walking speed on the diagonals reached a constant percentage of comfortable waking speed.

For the experimental protocol subjects were asked to walk at five different stride frequencies (columns in figure 3) in combination with five different stride lengths (rows in figure 3), expressed as a percentage of the comfortable values. This resulted in 25 trials of 4 minutes walking, all at an unique combination of stride frequency and stride length. The percentages of comfortable stride frequency and stride length were chosen in such a way that walking speed was the same for the trials on the diagonals from the upper left to the lower right in the experimental overview presented in figure 3. For each trial, treadmill speed was set at the required speed. Visual feedback on the required and the current combination of stride frequency and stride length was given as shown in figure 2. Subjects were instructed to keep the red dot as much as possible in the middle of the light gray cell. All trials were divided over two days and were offered in random order.

### Data collection

Kinematic data of markers attached at the lateral malleoli of the ankles, the heels and the cluster markers placed between the left and right PSIS were collected with the Optotrak system at a sampling rate of 100 samples/s.

### Data analysis

Before data analysis, except for the calculation of LDS, kinematic data were low-pass filtered with a bi-directional Butterworth filter with a cut-off frequency of 4 Hz. All parameters were calculated for the first 150 strides after the first 15 seconds of each trial.

#### Stride frequency and stride length

Stride frequency was determined as the inverse of the average duration between two subsequent heel-strikes, where heel-strikes were detected as the local maxima of the position of the ankle markers in the anterio-posterior direction. Stride length was calculated as the anterio-posterior distance between both ankle markers at the moment of heel-contact.

#### Margins of stability

To calculate the margins of stability, a method derived from the procedure developed by Hof [21] was used. In the present study, the extrapolated centre of mass (XCoM), was defined as the average of the three cluster markers attached between the left and right PSIS, as estimate for the centre of mass (CoM), plus its velocity times a factor √(l/g) , with l being the maximal height of the estimated CoM and g the acceleration of gravity. The margins of stability (MoS) were calculated as the position of the XCoM relative to the lateral malleolus of the ankle of the leading foot for the sideward MoS and relative to the heel marker of the leading foot for the backward MoS. For each step sideward and backward MoS were calculated for the moment at which the MoS reached its minimum value within each step and were subsequently averaged over 150 strides (300 steps). Although basically similar, our method differs from that of Hof [21] who used force plate data for calculating XCoM and margins of stability.

#### Local dynamic stability

To calculate LDS, position data of the cluster marker attached to the pelvis was used. Data were analyzed without filtering [31]. Linear velocities in the ML, AP, and vertical (VT) direction were calculated as the first derivative of the position of the average of the cluster marker. Time-series were time-normalized, using a shape-preserving spline interpolation, such that each time-series of 150 strides had a total length of 15,000 samples [10], [25], [32], [33]. Subsequently, 9D state spaces were reconstructed from the time-normalized 3D linear velocity time series, each with their 25 and 50 samples time delayed copies [32].

From the constructed state spaces, Euclidean distances between neighbouring trajectories in state space were calculated as a function of time and averaged over all original nearest neighbour pairs to obtain the average logarithmic rate of divergence. The slope of the resulting divergence curves for the interval between 0–50 samples provides an estimate of λs for the period of approximately 0–1 step [34], [35]. Larger values of λs imply that the LDS of the gait pattern is lower.

### Statistical analysis

To establish the relationship between stride frequency, stride length, and walking speed, all expressed as a fraction of the comfortable value, on the one hand, and the gait stability measures (ML and BW MoS, and the λs) on the other hand, Generalized Estimating Equations (GEE) were used. GEE is a regression analysis technique that accounts for the dependency of the repeated measurements. Because all trials were offered randomly, an exchangeable working correlation matrix was chosen to define this dependency of the repeated measurements in the model. With this technique it is possible to determine independently the contribution of stride frequency, stride length, and walking speed to the different outcome measures. This analysis was performed using IBM SPSS Statistics 20.0.

## Results

**Figure 4:**
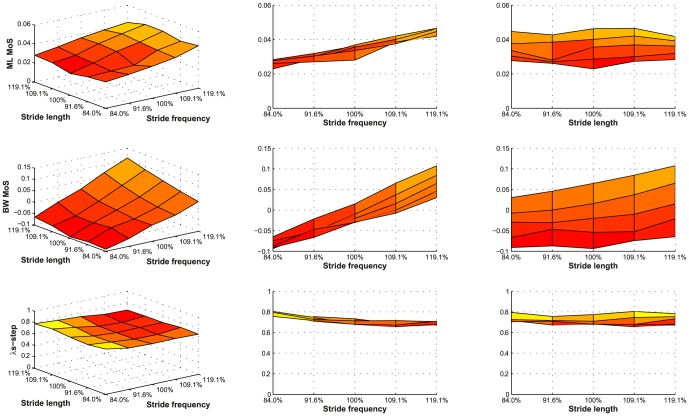
Results for ML MoS, BW MoS, and LDS. ML MoS (A), BW MoS (B), and λs (C) as a function of both stride length and stride frequency (left column). Light (yellow) areas represent a high value, while a dark (red) areas represent a low value for the concerning outcome measure. In the middle column the figure is oriented such that the relation between stride frequency and the outcome measure stands out. The same is done for stride length in the right column.

Figure 4 graphically depicts the results for ML and BW MoS, as well as λs, for all 25 experimental trials. The results of the GEE analyses are summarized in table 1. For ML MoS only a positive effect of an increase in stride frequency was found. BW MoS became larger as a result of a decrease in stride length and as a result of an increase in walking speed. For λs no significant effects of stride frequency, stride length or walking speed were found.

**Table 1:** GEE regression coefficients (β) for the effect of stride length, stride frequency and walking speed on the one hand and the ML MoS, the BW MoS and LDS expressed as λs on the other hand.

**ML MoS**:		
Parameter	β	p-value
Stride length	0.022	0.299
Stride frequency	0.068	<0.001*
Walking speed	−0.016	0.438
**BW MoS**:		
Parameter	β	p-value
Stride length	−0.321	0.009*
Stride frequency	−0.045	0.716
Walking speed	0.465	<0.001*
**LDS (λs)**:		
Parameter	β	p-value
Stride length	0.548	0.084
Stride frequency	0.288	0.331
Walking speed	−0.546	0.060

=  β_1_*stride length + β_2_*stride frequency + β_3_*walking speed + intercept In the model stride length, stride frequency, and walking speed are expressed as the fraction of the comfortable value. The model used is: outcome measure

*significant at the 0.05 level

## Discussion

The purpose of the present study was to investigate whether adaptations in stride frequency, stride length, and walking speed independently influence λs and ML and BW MoS, during walking. The results obtained contribute to the understanding of gait pattern selection in healthy and patient populations. Moreover they provide pointers for gait strategies that might be used in training programs aimed at fall prevention. We found that ML MoS increased with an increase in stride frequency, while adaptations of stride length and walking speed did not affect ML MoS. BW MoS increased with a decrease in stride length or an increase in walking speed, while stride frequency did not affect the BW MoS. Finally, none of the manipulations of stride frequency, stride length, and walking speed significantly influenced λs.

The increase in ML MoS with an increase in stride frequency and the increase in BW MoS with a decrease in stride length or an increase in walking speed were in line with our hypotheses. These results support the applicability of the theoretical models with respect to the MoS, in which the human body during walking is modeled as a simple inverted pendulum [21], [22], [23]. Furthermore, the results support previous observations that the increase in stride frequency and decrease in stride length found in several perturbation studies are strategies to increase ML and BW MoS, and thus to prevent a fall in the presence of perturbations[13], [15], [18].

The positive effects of a high walking speed and a small stride length on BW MoS may result in a conflict. Based on the representation of the results in figure 4 (right panel), we can conclude that the positive effect of the increase in walking speed on BW MoS outweighs the negative effect of the increase in stride length. This can also be derived from the larger regression coefficient (β) for walking speed, compared to stride length (table 1). For example, an increase in walking speed from 100% to 120% of comfortable walking speed will increase the BW MoS by 0.093 m. (1.2*0.465 compared to 1.0*0.465), while an increase from 100% to 120% of comfortable stride length will only decrease the BW MoS by 0.009 m. (1.2*-0.045 compared to 1.0*-0.045). So increasing walking speed enhances MoS even if it is achieved through an increase of stride length.

Remarkably, none of the manipulations of stride frequency, stride length, and walking speed had a significant effect on λs, although tendencies towards a significant increase in λs with an increase in step length, and a decrease in λs with an increase in walking speed were found. Besides, the regression coefficients (β) suggest that the effects of stride frequency and stride length are opposite to the effect of walking speed, which means that these effects might cancel each other when increasing or decreasing walking speed (table 1). Previous studies have shown that LDS, quantified as λs, did decrease as a result of external perturbations[13], [17], galvanic vestibular stimulation [33] or as a consequence of gait-impairments, like a lower-limb amputation [36]. However, the present results indicate that LDS does not change when people deviate from their comfortable gait pattern. The λs reflects the response to (small) perturbations within a period of one step after such a perturbation. This time period might be too short to allow influence on λs by adaptations of stride frequency and stride length which logically sort an effect after a period of a full stride. Consequently, λs might be more dependent on intrinsic stiffness [20] and gains of rapid feedback loops [33].

In comparing the present results on λs to the results of previous studies, the methodological choices made with respect to the calculation of λs have to be taken into account. The choices with respect to filtering and state space reconstruction might have influenced the results as different effects of gait speed and stride length on λs were previously found for different state space representations of trunk kinematics (i.e.[26], [37]). However, we have tested the effect of filtering of the data before calculating λs (10 Hz low-pass filter), and the effect of calculating λs separately for different planes (ML, AP and VT-plane, in line with McAndrew et al. [26]), and observed these alternative methods would not have influenced the conclusions drawn in the current study. These findings are in line with a previous study in which it appeared that the correlation between λs calculated from various state spaces was high [38]. Nevertheless, it should be taken into account that generalization of the conclusions drawn in the current study with respect to λs should be done keeping in mind these methodological issues.

For a proper interpretation of the results of this study, it is also important to note that our definition of MoS in anterior-posterior direction differ the from definition previously used by Hof [23]. Hof defined stability in anterio-posterior direction as the ability to maintain a more or less steady forward speed, while our definition for stability in anterio-posterior direction is the maintenance of forward progression. In the latter case the minimum criterion to maintain stability in anterio-posterior direction is that the XCoM passes the BoS during the double-support phase of the gait cycle, which might not be sufficient to minimize speed decrements and does not exclude speed increases. Secondly, one should be aware that the importance of increasing the BW MoS may strongly depend on the walking condition. In the case of, for example, stepping off a curb [39], or walking in a condition with a high risk of a forward trip [40], probably decreasing the risk of a forward fall, by increasing the forward MoS will be prioritized above increasing the BW MoS.

In conclusion, in line with the underlying mechanical models, ML MoS can be increased by an increase in stride frequency, and BW MoS can be increased by increasing walking speed or decreasing stride length. It appeared that when walking at a high speed, the positive effect of a high speed on BW MoS outweighs the negative effect of the large stride length employed to reach this high walking speed. When walking speed is limited, for example by energetic constraints, walking with fast and short steps at a certain walking speed results in the largest ML and BW MoS. LDS was not significantly affected by adaptations in stride frequency, stride length, and walking speed. Based on these results, we conclude that adaptations in stride frequency, stride length, and walking speed can be used to increase ML and BW MoS, without a loss of LDS (expressed as λs), when gait stability is challenged. Increases in ML and BW MoS may thus compensate for a potential decrease in LDS, caused by external perturbations [13], [17] or as a consequence of a gait-impairment [36], in order to prevent an actual loss of balance. Besides, the results of the current study are an indication that the slower walking speed, with a lower stride frequency and a smaller stride length, often employed by people with gait impairments [5], [6], [7], [8], may be a cause of an increased fall risk, instead of a strategy used to minimize fall risk. Therefore, it would be of interest to investigate whether people with gait impairments are able to walk at different combinations of stride frequency and stride length, and how these alterations affect the MoS. Training focused on the adaptation in stride frequency and stride length at different walking speeds might help these people to better regulate gait stability, and therewith decrease their risk of falling.
